# Long-lasting effects of World War II trauma on PTSD symptoms and embodiment levels in a national sample of Poles

**DOI:** 10.1038/s41598-023-44300-6

**Published:** 2023-10-11

**Authors:** Marcin Rzeszutek, Małgorzata Dragan, Maja Lis-Turlejska, Katarzyna Schier, Paweł Holas, Małgorzata Pięta, Angelika Van Hoy, Katarzyna Drabarek, Cecylia Poncyliusz, Magdalena Michałowska, Gabriela Wdowczyk, Natalia Borowska, Szymon Szumiał

**Affiliations:** 1 Faculty of Psychology, University of Warsaw, Stawki 5/7, 00-183 Warsaw, Poland; 2 Faculty of Psychology, SWPS University of Social Sciences and Humanities, Chodakowska Street 19/31, 03-815 Warsaw, Poland

**Keywords:** Psychology, Post-traumatic stress disorder, Trauma

## Abstract

The main aim of this study was to investigate the long-lasting influences of World War II (WWII) trauma in a national sample of Poles, based on Danieli’s (1998) survivors’ post-trauma adaptational styles (fighter, numb, victim) and their link with current post-traumatic stress disorder (PTSD) symptoms and embodiment level among participants. We also sought to investigate whether the level of knowledge about WWII trauma among ancestors could moderate that association. The study was conducted among a representative sample of 1598 adult Poles obtained from an external company. Participants filled out the Danieli Inventory of Multigenerational Legacies of Trauma, the knowledge about traumatic World War II experiences in the family questionnaire, the Posttraumatic Diagnostic Scale-5, and the Experience of Embodiment Scale. We observed a positive relationship between all survivors’ post-trauma adaptational styles and current levels of PTSD symptoms among participants. In addition, PTSD level mediated the relationships between those adaptational styles and embodiment intensity; that mediation was additionally moderated by a lack of knowledge about WWII trauma among ancestors in our participants. Our study adds to the literature on intergenerational trauma by highlighting the importance of evaluating embodiment in understanding the mechanisms of trauma transmission. Furthermore, it highlights the moderating effect of knowledge of family history in this mechanism and the need to share family histories with subsequent generations.

## Introduction

It has been more than half a century since Rakoff et al.^1^ observed the problem of *intergenerational trauma* among descendants of Holocaust survivors who did not experience the Holocaust directly but were secondarily traumatized by parental traumatic history and the related negative emotional atmosphere in their family of origin^2^. More specifically, numerous subsequent studies have found that Holocaust offspring were at increased risk of mental health problems, predominantly posttraumatic stress disorder (PTSD), depression, and anxiety^2–6^, as well as heightened prevalence of health problems like cancer, heart disease, and chronic pain^7–10^. These studies on second-generation Holocaust survivors started a new, interdisciplinary line of research focused on whether and how the consequences of surviving trauma may be transmitted from one generation to another (for a review, see^11^).

The term *transmission of trauma* has been defined in various ways^11^ but was originally understood as the process of passing specific, trauma-related thoughts, feelings, and behaviors from trauma survivors to their offspring^5,12^. This process has been observed not only in the case of Holocaust trauma but also among children of war veterans^13^, families of refugees^14^, and even families in which one parent was exposed to childhood maltreatment and abuse^15^. Various mechanisms for such trauma transmission processes have been suggested, including psychodynamic explanations^16^, vicarious trauma models^17^, learning and modeling^13^, parenting and family environment influences^18,19^, and even biological hypotheses^10,20^. Although the literature on intergenerational trauma is massive and still expanding (for reviews and meta-analyses, see^11,21,22^, the topic remains rife with controversies and unanswered but important research questions. First, there is little knowledge of how trauma is transmitted from generation to generation^11^, and several authors have found no evidence that trauma transmission occurs^23^. Second, most studies on the subject employ small convenience samples of trauma survivors and draw exaggerated conclusions, often devoid of any theoretical rationale^11^. Finally, in Europe especially, research on intergenerational trauma is unevenly distributed geographically, with few studies focused on Central and Eastern Europe^24^. In our project, we aimed to fill some of these research gaps by investigating the long-lasting influences of World War II (WWII) trauma in a national sample of Poles based on the theoretical model of multigenerational legacies of massive trauma: *Trauma and the Continuity of Self: A Multidimensional, Multidisciplinary, Integrative Framework* (TCMI) by Danieli^2^.

Extensive empirical research and clinical experience working with prolonged and complex traumatic events (predominantly Holocaust survivors) led to the TCMI framework, illustrating the multidimensional and intergenerational nature of surviving massive trauma and adapting to life's circumstances in its aftermath^2,25,26^. TCMI operationalizes the trauma survivor's identity at multiple levels: intrapsychic, family, community, national, and cross-cultural^2,25,26^. Traumatic experiences cause serious ruptures on all levels. Often, this process is further exacerbated by a conspiracy of silence surrounding such trauma within an individual's immediate social circle. As a result, the trauma survivor is forced to adopt specific survival strategies, called *survivors' posttrauma adaptational styles*, which are classified into three groups: *victim* (being stuck in the trauma rupture, emotional volatility, and overprotectiveness), *numb* (characterized by emotional detachment, conspiracy of silence within the family, and intolerance of weakness), and *fighter* (illustrated by valuing and maintaining national identity and cherishing mastery and justice). These adaptational styles are essentially ways to cope with massive traumatic events and are adopted by those who directly experience them. However, they also impact trauma survivors' family members by structuring their identity, emotions, and beliefs about themselves, society, and the world in general^2,26^. Some empirical studies suggest that parents' intergenerational influences on these adaptational styles affect their offspring's psychosocial development, increasing susceptibility to PTSD symptoms following exposure to a traumatic event^19,27^. In our study, we investigate the role of Danieli's survivors' posttrauma adaptational styles to understand the long-lasting impacts of WWII trauma in Poland. The psychosocial effects of WWII remain an unspoken topic of scientific discourse and public debate in Poland^28,29^. However, a few studies have revealed a significant discrepancy between the prevalence of WWII-related PTSD among survivors of the war in Poland compared to other European countries. For example, the prevalence of PTSD among civilian survivors of WWII ranged from 1.9% in Austria^30^ to 10.9% in Germany^31^, while PTSD among Polish survivors of that war has been reported to range from 29.4%^32^ to 38.3%^29^.

Apart from the fact that the observed differences can be related to differences in the measurement methods, this discrepancy is explained by historical factors associated with the vast human and material losses experienced by Poles during WWII, along with repression and insecurity during the Communist regime^33,34^. In other words, Polish survivors of WWII could face powerful obstacles in revealing their traumatic memories in their communities. Thus, they received inadequate support and social acknowledgment of their traumatic WWII experiences^29^. They might transfer this atmosphere of anxiety and taboo to their offspring^35^. Some authors have shown that the transmission of intergenerational trauma in families happens through specific structures of attachment and communication^36–38^. This aligns with developmental theories, highlighting that sharing a family history with children significantly predicts their self-identity and psychological well-being^39^. Nevertheless, there is still scant research on the issue of knowledge about traumatic family stories, particularly related to the experiences of ancestors^40,41^.

Finally, we focus on the role of body image as operationalized by the level of embodiment highlighting a variety of experiences of living in one's own body; that is, referring to an integrated set of connections in which a person experiences their body as comfortable, trustworthy, and deserving of respect and care^42–44^. Traditionally, psychotherapy underscores the role of appropriate orientation toward body signals as an integral part of the self, which helps individuals sustain their mental and physical health, particularly in the face of stress and trauma^45^. A large body of empirical research has since shown how the experience of one's own body can be significantly hampered by experiences of traumatic events^46,47^ and how body-oriented therapy can help trauma survivors in dealing with PTSD (for a metanalysis, see^48^). Theoretical explanations of this process have usually focused on a deterioration in the orientation toward body signals in the aftermath of PTSD symptoms that can result in applying a catastrophic or alien orientation toward the body and experiencing one's body as detached from one's self^49–51^. Importantly, similar negative bodily experiences have been observed among offspring of Holocaust survivors^8^ and children of refugee families^14^, showing that the intergenerational effects of trauma emerge in multidimensional rather than singular psychosocial outcomes^11^.

### The present study

Taking the above considerations into account, the primary objective of this study was to explore the long-term effects of WWII trauma on a national sample of Polish people. We analyzed survivors' posttrauma adaptational styles (victim, numb, fighter) based on Danieli's^2^ framework. We also examined the potential correlation between their adaptational styles and present-day PTSD symptoms and embodiment. Additionally, we investigated how the participants' upbringing in the shadow of WWII trauma, family atmosphere, and reflections on their parents or grandparents who experienced WWII trauma influenced their posttrauma adaptational styles. Finally, we aimed to investigate whether a lack of knowledge about WWII trauma among their ancestors could moderate these relationships. Although our study is exploratory to a large extent regarding the study sample, study context, and the configuration of study variables, we decided to formulate research hypotheses that direct our analyses:

#### *Hypothesis 1*

There is a significant positive relationship between posttrauma adaptational styles (victim, numb, fighter) and the current PTSD symptom levels among participants while controlling for sociodemographic factors. More specifically, we assume that those posttrauma adaptational styles can also be seen in succeeding generations and have the associations noted above with current PTSD symptoms.

#### *Hypothesis 2*

There is a significant negative association between current levels of PTSD symptoms and levels of embodiment among participants while controlling for sociodemographic factors.

#### *Hypothesis 3*

Current levels of PTSD symptoms are a significant mediator between survivors' posttrauma adaptational styles (fighter, numb, victim) and embodiment levels among participants while controlling for sociodemographic factors.

#### *Hypothesis 4*

A lack of knowledge about WWII trauma among ancestors significantly moderates the mediation process noted in Hypothesis 3 while controlling for sociodemographic factors among participants.

## Method

### Participants and procedure

A survey was conducted in September and October 2022 by an external company specializing in nationwide Polish research panels among a representative sample of 1598 adult Poles aged 18–97 (M = 48.78; SD = 20.50). Participants represented different generations of Poles born before, during, and after WWII (see Table 1). The external company used its survey platform to distribute an online version of the measures. Participation in this study was entirely anonymous and voluntary. Each participant provided informed consent, and the survey company provided remuneration by granting specific tokens. This research project was accepted by the ethics committee of the Faculty of Psychology at the University of Warsaw in Poland.

**Table 1:** Sociodemographic characteristics of the study sample.

Sociodemographic characteristics	*n*	%	
Gender	Female	810	50.7
	Male	788	49.3
Age in years	18–46	788	49.3
	47–76	649	40.6
	> 77	161	10.1
Place of residence	Village	555	34.7
	Small town (< 20,000 residents)	193	12.1
	Medium town (20,000–99,000 residents)	313	19.6
	Larger city (100,000–500,000 residents)	293	18.3
	Big city (> 500,000 residents)	244	15.3
Education	Primary	39	2.4
	Vocational	128	8.0
	Secondary	645	40.4
	Incomplete higher	80	5.0
	Higher	706	44.2
Relationship status	Single	320	20.0
	Married	794	49.7
	Informal relationship	239	15.0
	Separated	18	1.1
	Divorced	81	5.1
	Widowed	146	9.1

*n* Number of participants.

Table 1 presents the sociodemographic characteristics of the study sample.

The numbers of females (50.7%) and males (49.3%) were similar, and most participants were aged 18–46. The most commonly identified place of residence was a village. Most participants had higher education (44.2%) and were married (49.7%).

### Measures

*Survivors' posttrauma adaptational styles*. To assess the multigenerational effect of WWII trauma among our participants, we used the Polish version of the *Danieli Inventory of Multigenerational Legacies of Trauma: Survivors' Posttrauma Adaptational Styles*^18^*.* This tool is considered the gold standard for examining the multigenerational effects of massive traumas (e.g., Holocaust, war, genocide). It consists of 60 items describing specific emotions, beliefs, or behaviors evaluating people's perceptions of their parents and grandparents and upbringing as a reaction to such traumas in a family's history. The three adaptational styles described above—victim, numb, and fighter—are measured via this tool. Participants used a five-point Likert scale to rate their level of agreement with statements about the atmosphere and communication styles in their family of origin. We instructed participants to focus on the context of WWII trauma among their ancestors and the associated behaviors of their parents or grandparents towards them, depending on the age of the study respondents. The psychometric properties of the inventory were satisfactory and are presented in Table 2.

**Table 2:** Descriptive statistics acquired in the study sample.

Variables	*M*	*SD*	*Min*	*Max*	*S*	*K*	*alpha*	*omega*	Pearson’s correlation coefficients				
									1	2	3	4	5
1. Fighter	2.82	0.51	1	5	0.02	1.51	0.65	0.65	–	–	–	–	–
2. Numb	2.61	0.51	1	5	0.25	1.34	0.76	0.77	0.62**	–	–	–	–
3. Victim	2.56	0.58	1	5	0.17	0.76	0.91	0.92	0.70**	0.87**	–	–	–
4. Lack of knowledge of family’s WWII trauma	18.11	28.78	0	108	1.76	2.16	–		− 0.15**	− 0.01	− 0.04	–	–
5. Total PTSD symptoms	16.36	15.93	0	80	1.13	0.78	0.97	0.97	0.30**	0.41**	0.49**	− 0.012	−
6. Embodiment	116.88	18.51	50	164	0.11	0.08	0.93	0.93	− 0.05	− 0.30**	− 0.32**	− 0.11**	− 0.46**

*M* Mean value; *SD* Standard deviation; *Min* Minimum value; *Max* Maximum value; *S* Skewness; *K* Kurtosis;

*alpha*, Cronbach’s alpha internal consistency coefficient; *omega*, McDonald’s omega internal consistency coefficient; ** *p* < .01.

#### Knowledge of traumatic WWII experiences in the family

This inventory checked participants' knowledge of traumatic events that their parents and/or grandparents (depending on participant age) may have experienced during WWII^41^ (see Appendices). Participants were asked twenty-seven questions associated with various WWII-related traumatic experiences of their ancestors. More specifically, respondents rated whether they knew whether a given event had or had not occurred in the lives of their parents and/or grandparents or whether they did not know. The number of events not known by the participants, which indicates a lack of knowledge of WWII trauma in the family of origin, was then calculated as the moderator variable in our analyses (see data analysis and results).

#### Current PTSD symptoms

To measure current PTSD symptoms among study participants, we used the Polish adaptation of the Posttraumatic Diagnostic Scale-5 (PDS-5), a 24-item self-report measure investigating the severity of PTSD symptoms using criteria from the fifth edition of the Diagnostic and Statistical Manual of Mental Disorders, Fifth Edition^52^, (DSM-5). We followed the total PTSD symptom score and instructed study participants to complete the PTSD section about the main aim of the present study. The psychometric properties of this inventory were also satisfactory and are presented in Table 2.

#### Level of embodiment

To evaluate the construct of embodiment among participants, we used the Polish adaptation of the Experience of Embodiment Scale^53^ (EES), which consists of six subscales assessing various components of embodiment on a five-point Likert scale: positive body connection and comfort, body unencumbered adjustment, agency and functionality, experience and expression of sexual desire, attuned self-care, and resisting objectification. In this study, we followed the global embodiment indicator and instructed participants to complete this tool with regard to our main research aim. The psychometric properties of this tool were also satisfactory and are presented in Table 2.

### Data analysis

**Figure 1:**
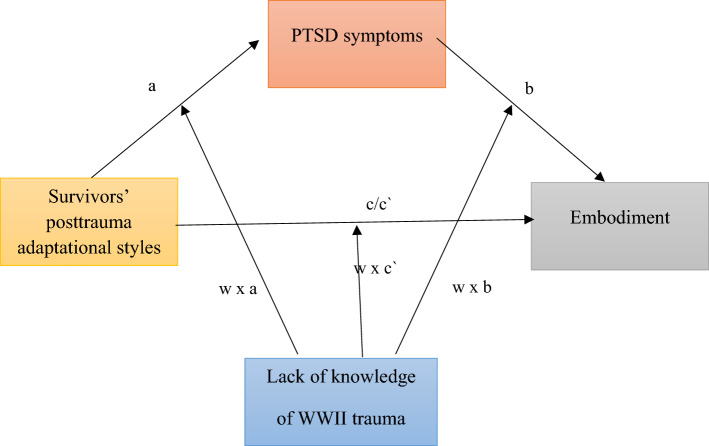
Analyzed model of relationships between variables.

The results were analyzed within the framework of moderated mediation, using PROCESS Macro v. 4.3^54^. The adaptational styles of victim, numb, and fighter were analyzed as explanatory variables, with the intensity of current PTSD symptoms investigated as a mediator. Embodiment was examined as an explained variable, and lack of knowledge about WWII trauma was analyzed as a moderator. Figure 1 depicts the study's analytical plan. Participant age, gender, place of residence, education, and relationship status were included in the models as covariates to control for their impact on the analyzed processes.

### Ethical approval

All procedures performed in studies involving human participants were in accordance with the ethical standards of the institutional and/or national research committee and with the 1964 Declaration of Helsinki and its later amendments or comparable ethical standards.

## Results

Table 2 presents descriptive statistics for the interval variables in the current study: mean values, standard deviations, minimum and maximum values, measures of skewness and kurtosis, and Cronbach's alpha internal consistency coefficients, McDonald’s omega internal consistency coefficients and Pearson’s correlation matrix.

No significant deviations from a normal distribution were detected, except for the lack of knowledge, the distribution of which was positively skewed and leptokurtic. The scores on the Fighter, Numb, and Victim scales were positively correlated with each other and with global PTSD symptoms intensity. The scores on the Numb and Victim scales were negatively correlated with the level of embodiment. Global PTSD symptoms intensity was negatively correlated with the level of embodiment. The lack of knowledge of the family’s WWII trauma correlated negatively with the scores on the Fighter scale and the embodiment level. However, these two negative correlations were very weak.

Of the five control variables, only participant age was significantly related to the main variables: it correlated negatively with the level of PTSD (*B* (95% CI) = [− 0.02; − 0.01], *p* < 0.001) and positively with the level of embodiment (*B* (95% CI) = [0.01; 0.02], *p* < 0.001). Thus, increased age correlated with less intensive PTSD symptoms and better levels of embodiment. The results of the moderated mediation analysis are presented in Table 3. Three separate models were calculated for the three posttrauma adaptational styles. The a, b, c`, and w symbols match Fig. 1 inserted above.

**Table 3:** The results of moderated mediation analysis: Total PTSD symptoms as a mediator of the relationship between survivors’ posttrauma adaptational styles and embodiment levels as moderated by a lack of knowledge of WWII trauma.

IV	a	w x a	b	w x b	c`	w x c`	R^2^
Fighter	[0.24; 36]***	[− 0.09; 0.03]	[− 0.50; − 0.38]***	[− 0.12; 0.02]	[0.01; 0.12]	[− 0.14; − 0.02]**	0.26
Numb	[0.31; 43]***	[− 0.12; 0.01]	[− 0.41; − 0.29]***	[− 0.15; − 0.02]*	[− 0.24; − 0.12]***	[− 0.06; 06]	0.28
Victim	[0.39; 0.51]***	[− 0.14; − 0.02]**	[− 0.42; − 0.29]***	[− 0.14; − 0.01]*	[− 0.22; − 0.09]***	[− 0.09; 0.04]	0.27

The table presents 95% CI intervals for standardized regression coefficients.

A, b, c` and w symbols match Fig. 1; IV = independent variable; *R*^2^ = determination coefficient. *p < 0.05; **p < 0.01; ***p < 0.001.

All three posttrauma adaptational styles were positively related to the intensity of PTSD symptoms (path a), which supports Hypothesis 1. In addition, the level of PTSD symptoms was negatively associated with the level of embodiment in all three analyzed models (path b), confirming Hypothesis 2. Moreover, PTSD symptoms mediated the relationships between posttrauma adaptational styles and levels of embodiment in all three adaptational styles: fighter (*B* (95% CI) = [− 0.18; − 0.10]), numb (*B* (95% CI) = [− 0.17; − 0.10]), and victim (*B* (95% CI) = [− 0.20; − 0.12]). Higher levels of the adaptational styles were connected with higher levels of PTSD symptoms (path a), leading to lower levels of embodiment (path b). These results confirm Hypothesis 3.

There were also statistically significant interactions between lack of knowledge about WWII trauma and adaptational style and between lack of knowledge about WWII trauma and PTSD symptoms in the models in which the numb and victim adaptational styles were analyzed as independent variables and between lack of knowledge about WWII trauma and the fighter adaptational style in relationship to the level of embodiment. These interaction effects confirm Hypothesis 4. In order to interpret this moderation, conditional direct and indirect effects were calculated.

It was found that the direct positive relationship between the fighter adaptational style and embodiment level was statistically significant only if the degree of knowledge was average (i.e., close to the mean value in the current sample: *B* (95% CI) = [0.04; 0.18], *p* < 0.01), or one standard deviation below the average (*B* (95% CI) = [0.04; 0.18], *p* < 0.01. If the lack of knowledge was high (i.e., greater than one standard deviation above the average), there was no direct relationship between the fighter adaptational style and levels of embodiment (*B* (95% CI) = [− 0.06; 0.08], *p* > 0.05).

In addition, in the model analyzing the numb adaptational style as an independent variable, it was found that the negative relationship between the intensity of PTSD symptoms and level of embodiment was stronger if the lack of knowledge was at least one standard deviation above the average (*B* (95% CI) = [− 0.48; − 0.33], *p* < 0.001). This relationship was weaker if the lack of knowledge was average (*B* (95% CI) = [− 0.37; − 0.22], *p* < 0.001) or one standard deviation below the average (*B* (95% CI) = [− 0.37; − 0.22], *p* < 0.001).

In the model analyzing the victim adaptational style as an independent variable, it was found that the positive relationship between the victim adaptational style and PTSD symptoms was weaker if the lack of knowledge was at last one standard deviation above the average (*B* (95% CI) = [0.33; 0.47], *p* < 0.001). This relationship was stronger if the lack of knowledge was average (*B* (95% CI) = [0.44; 0.57], *p* < 0.001) or at least one standard deviation below the average (*B* (95% CI) = [0.44; 0.57], *p* < 0.001). It was also found that the negative relationship between the intensity of PTSD symptoms and embodiment level was stronger if the degree of knowledge was at least one standard deviation above the average (*B* (95% CI) = [− 0.24; − 0.09], *p* < 0.001). This relationship was weaker if lack of knowledge was average (*B* (95% CI) = [− 0.21; − 0.06], *p* < 0.01) or less than the standard deviation below the average (*B* (95% CI) = [− 0.21; − 0.06], *p* < 0.01).

## Discussion

The results of our study support hypothesis 1 (H1), as we observed a relatively strong and positive relationship between all three survivors' posttrauma adaptational styles (fighter, numb, victim) and current levels of PTSD symptoms among participants. In other words, although our findings should be treated with caution due to their correlational nature, they may indicate the potential long-term impact of intergenerational trauma transmission through greater susceptibility to PTSD symptoms after trauma exposure^55,56^, particularly a higher predisposition to lifelong PTSD among succeeding generations^4^. Research on Holocaust offspring conducted in community samples showed that they reported higher rates of PTSD and affective and anxiety disorders than Jewish controls^5^. Danieli^2^ described the process of specific negative identity formation among offspring of Holocaust survivors about themselves and the world in general, which makes them significantly less resilient to stress and adversity throughout the life cycle. Recent studies underlined the process of the intergenerational transmission of specifically war-related PTSD in families. However, this topic remains controversial regarding the pathways by which such transmission occurs (for a review, see^57^).

In our study, we concentrated on one possible pathway relating to the participants' experiences of their bodies as operationalized by the level of embodiment. To achieve this research aim, we formulated and confirmed two additional hypotheses (see Fig. 1), which we verified positively to a large extent. More specifically, on the one hand, we observed that the level of PTSD symptoms was negatively linked to the level of embodiment in all three analyzed models. On the other hand, the level of PTSD mediated the relationships between posttrauma adaptational styles and intensity of embodiment, again in all three adaptational styles we investigated. In other words, the possible long-term effects of intergenerational transmission of WWII trauma in the families of our participants were revealed in poor levels of embodiment that were exacerbated by co-occurring PTSD symptoms. Efforts to explore the relationship between PTSD and embodiment should be grounded in a minimum of two distinct theoretical frameworks. First, the allostatic load theory illustrates how traumatic events experienced in childhood may influence somatic health in adulthood^58^. Specifically, allostasis is defined as the ability of the body to adjust to varying external or internal factors to maintain stable physiological states. Chronic overactivation of the nervous, endocrine, or immune systems due to exposure to psychological trauma causes what is known as the allostatic load. Danese and McEwen^59^ found that chronic stress during childhood interferes with the deterioration of those three physiological systems, leading to a decline in physical health and various somatic problems. A study of the relationship between WWII trauma and physical and mental health in the German population recently demonstrated the foundations of this theory^60^. Second, the most common symptoms of PTSD, as a possible reaction to highly stressful events, are related to physiological hyperarousal and altered reactivity at exposure to trauma-related cues. They are linked to substantial dysregulation of arousal and affect modulation (impaired self-regulation), somatization, and problems with body awareness^48,61^. These difficulties are observed in studies based on self-reports and research on the biology of PTSD, indicating differences in posttrauma stress reactivity and brain alterations reflecting emotional dysregulation^62,63^. According to van der Kolk^46,47^, trauma is "written down in the body"; his theory on somatic memory of trauma highlights that it may translate in trauma survivors into a process of distrusting signals from one's own body. This mechanism may explain why trauma survivors often develop a catastrophic orientation towards their bodily signals, suffer from impairment in orientation to the body and its boundaries, and, in turn, experience low levels of embodiment^38,53,64^.

Our project aimed to test the moderating effect of the degree of knowledge about WWII trauma among ancestors on the mediated mechanism of the transmission of WWII intergenerational trauma among participants. Interestingly, in all three models of posttrauma adaptational styles, a lack of knowledge about WWII-related traumatic history in the family of origin translated into a higher intensity of PTSD symptoms and, thus, poorer levels of embodiment (see Fig. 1 and Table 3). In addition, what was also surprising and counterintuitive was that out of all sociodemographic variables, only the participants' age was significantly related to the main variables but correlated negatively with the level of PTSD and positively with the level of embodiment. Simply put, the transmission of WWII trauma appears to have affected the younger generations more than the older ones. The first generation had first-hand experience of the various traumatic events during the war, while those who came after them were more psychologically impacted by the aftermath of the war. This result may be better understood within the TCMI framework and the construct of a *conspiracy of silence* concerning intergenerational trauma^2,18,19,27^. According to this view, massive and complex trauma forces a family to keep silent about it. It is an impulse that arises from two sometimes conflicting factors: willingness to protect other family members from the family's unbearable traumatic past and the problems trauma survivors have in revealing their traumatic stories to family members, which might be linked to trauma denial or a lack of social acknowledgment of their traumatic memories. All these processes may contribute to the deterioration of the family system, leading to the transmission of trauma to subsequent generations^65,66^. One should remember that families who shared WWII-related traumatic experiences may also form more intimate relationships with family members, which may translate into better psychological well-being. All in all, it appears that only sharing the entire family history with offspring, no matter how traumatic that history is and how difficult it may be to share it, can help put an end to the vicious cycle of intergenerational transmission of trauma, as has been highlighted by recent work^19,40,41^.

### Strengths and limitations

Our research has numerous strengths, including the theory-driven design of a study conducted on a large and representative sample of Poles. Nevertheless, certain limitations should be noted. First, the Danieli inventory was originally constructed in the context of Holocaust trauma. Although we consulted on the process of modifying this tool for the context of WWII trauma with the author, the results could still be biased. Second, although we instructed study participants to complete the PTSD and embodiment questionnaires in reference to the main aim of this study—WWII trauma among their ancestors—those questionnaires were not directly created for that purpose. Thirdly, not all of the scales in the Polish adaption of the Danieli Inventory had equally satisfactory reliability (the fighter subscale). Lastly, our study is correlational and cross-sectional, which precludes causal inferences and shares the drawbacks typical of research on intergenerational trauma^21^, in which prospective studies are incredibly scarce due to organizational and ethical reasons^67^.

## Conclusion

Despite these limitations, our study makes an essential contribution to the research on intergenerational trauma. First, it helps address the research gap regarding the link of embodiment and trauma transmission across generations, mediated by posttraumatic stress symptoms. Second, it highlights the moderating effect of the level of knowledge of family history in this mechanism and the need to share family histories with offspring for the sake of the psychological well-being of younger generations. Finally, our findings can be a starting point for more empirical research on the long-lasting effects of WWII trauma in Poland.

### Supplementary Information

Supplementary Information 1.

Supplementary Information 2.

## Data Availability

All data generated or analysed during this study are included in this published article (and its Supplementary Information files).
